# Supplementary material for: L-CPPA: Lattice-based conditional privacy-preserving authentication scheme for fog computing with 5G-enabled vehicular system
Source: PLoS One. 2023 Oct 27;18(10):e0292690. doi: 10.1371/journal.pone.0292690 (PMC10610142; doi:10.1371/journal.pone.0292690)
Supplement: Attachment — Submitted filename: Response to Reviewers.docx [file pone.0292690.s001.docx]

Original Manuscript ID: PONE-D-23-11761

Original Article Title: “L-CPPA: Lattice-Based Conditional Privacy-Preserving Authentication Scheme for Fog Computing with 5G-Enabled Vehicular System”

To: PLOS ONE

Re: Response to reviewers

Dear Editor,

Thank you for allowing a resubmission of our manuscript, with an opportunity to address the reviewers’ comments.

Best regards,

Dr. Mahmood A. Al-Shareeeda.

Reviewer 1: Concern #1:. Performance in terms of percentage must be mentioned in the abstract.

Reviewer 1: Response #1: Thanks for the valuable comments. We revise the manuscript by adding the result in terms of computation cost and communication costs at the end of the abstract as follows.

“Finally, the computation cost of the proposed L-CPPA scheme in terms of message signing, single verification and batch verification is 694.161 ms, 60.118 ms, and 1348.218 ms, respectively. Meanwhile, the communication cost is 7757 bytes.”

Reviewer 1: Concern #2:. To make the paper more readable and the results more convincing, the significance of the paper should be clearly explained relative to previous work. Besides, several of the critical background literature especially for fog computing and privacy-preserving are missing including but not limited to "A provably secure key transfer protocol for the fog-enabled Social Internet of Vehicles based on a confidential computing environment" and "Privacy‐preserving authentication scheme for digital twin‐enabled autonomous vehicle environments".

Reviewer 1: Response #2: Thanks for the valuable comments. We revise the manuscript by adding these two relevant articles for defining fog computing and privacy-preserving in the introduction section as follows.

“Information overflow due to redundant data may occur in autonomous vehicles because of the necessity for real-time sensing, calculation, and communication. In addition, the limited range of current communication technologies makes it challenging to anticipate traffic conditions outside of a vehicle's line of sight. Autonomous driving conditions have seen the development of digital twin systems to address these concerns [9]. Sensitive information about drivers is easily intercepted and altered since the data created by automobiles in motion with servers is carried on public networks. Furthermore, servers are put under extreme strain by the vast quantities of real-time data produced by these vehicles, devices, drivers, passengers, and other social links. As a result, Chen et al. [10] presented a secure authentication technique that makes use of private cloud infrastructures. In addition, they provided a more efficient key transfer phase to lessen the burden of computation on cloud servers.”

Reviewer 1: Concern #3. The authors should provide an adversary model.

Reviewer 1: Response #3: Thanks for the valuable comments. We revise the manuscript by providing an adversary model in the section of preliminaries as follows.

This subsection defines the scope and limitation of the adversary model used in the proposed L-CPPA scheme for fog computing with a 5G-enabled vehicular system. The proposed LC-CPPA scheme should be resisted the following adversary model.

- Modify Assaults: A robust policy against impersonation attacks is one that effectively stops attackers from posing as legitimate users.

- Man-In-The-Middle (MITM) Assaults: To resist MITM, a third party should be not able to capture the message sent between the legal senders and receivers.

- Forgery Assaults: To prevent a forgery attack, the protocol must be able to expose the attacker's effort to fabricate the sent traffic.

- Replay Assaults: To prevent traffic from being recovered from one session and used in another, the protocol should incorporate a nonce or timestamp into each message.

- Quantum Assaults: To resist quantum attacks, traditional cryptography should be not used in the proposed.

Reviewer 1: Concern #4:. The conclusion section is not impressive. This section should include a very brief discussion on future works. What are the problems that go beyond the scope of this paper? What are the issues that need to be addressed in contemporary research?

Reviewer 1: Response #4: Thanks for the valuable comments. We revise the manuscript by adding future work in the conclusion section as follows.

“In future work, we carry out experiment results using simulation networks such as OMNeT++ and traffic simulations such as SUMO. Meanwhile, this paper will extend to protect TA masquerading attacks.”

Reviewer 1: Concern #5:. A more thorough proofreading of this work would be beneficial.

Reviewer 1: Response #5: Thanks for the valuable comments. The manuscript has been proofread by a professional English service.

----------------------------------------------------------------------------------------------

Reviewer 2: Concern #1:. The proposed scheme is weak against TA masquerading attack by registered fog servers because TA preloads its master secret key E to all fog servers.

Reviewer 2: Response #1: Thanks for the valuable comments. We revise the manuscript by providing the adversary model applied for this proposal and adding the assumption in the system model.

*In a subsection of the Adversary Model,

This subsection defines the scope and limitation of the adversary model used in the proposed L-CPPA scheme for fog computing with a 5G-enabled vehicular system. The proposed LC-CPPA scheme should be resisted the following adversary model.

- Modify Assaults: A robust policy against impersonation attacks is one that effectively stops attackers from posing as legitimate users.

- Man-In-The-Middle (MITM) Assaults: To resist MITM, a third party should be not able to capture the message sent between the legal senders and receivers.

- Forgery Assaults: To prevent a forgery attack, the protocol must be able to expose the attacker's effort to fabricate the sent traffic.

- Replay Assaults: To prevent traffic from being recovered from one session and used in another, the protocol should incorporate a nonce or timestamp into each message.

- Quantum Assaults: To resist quantum attacks, traditional cryptography should be not used in the proposed.

*Besides, in a subsection of the proposed system model, under TA, (In this paper, we assume the TA component is very strong and reliable from masquerading attacks.).

Reviewer 2: Concern #2:. It needs to clarify how a vehicle use ID and password.

Reviewer 2: Response #2: Thanks for the valuable comments. We revise the manuscript by clearly explaining how we use vehicle ID and password in the proposed L-CPPA scheme section as follows.

During the registration step, the driver inputs the vehicle TID and Password PWD to TA for receiving system parameters and verification code. With this step, the vehicle is able to join the vehicular system to complete the next step as follows. Vehicles hide true identity TID by hashing verification codes to obtain anonymous identity AID, which uses publicly to exchange the message among vehicles or nearby fog servers via 5G-BS. While during message signing, the vehicles hide TID by hashing the verification code and current timestamp together. TID and AID refer to true identity and anonymous identity, respectively, which describes in Table 1.

Reviewer 2: Concern #3:. Cryptographic scheme design should be clearly defined every notation before they are used. However, there are lots of mistakes on the notations usage. Furthermore, the functions used in this paper should be clearly defined.

Reviewer 2: Response #3: Thanks for the valuable comments. We revise the manuscript by double-checking the notation usage and clearly defining the notation used in Table 1 as follows.

Reviewer 2: Concern #4:. It is unclear that how Ci should be the same as D*SKi.

Reviewer 2: Response #4: Thanks for the valuable comments. For more detail about the algorithm of SampleD, please see paper entitled (Trapdoors for hard lattices and new cryptographic constructions)

Reviewer 2: Concern #5:. The scheme does not provide freshness of message because it uses the same SKi for each session.

Reviewer 2: Response #5: We would like to clarify the concern as follows. In the message signing step, the vehicle multiplies Ski hashing value that included the anonymous-ID and current timestamp to obtain the signature key Pki, which each session has different timestamps for providing freshness message. Meanwhile, the verifier also checks the timestamp before accepting the messages.

Signer side:

Verifier side:

Reviewer 2: Concern #6: There are a lot of mistakes on the notation usages.

Reviewer 2: Response #6: Thanks for the valuable comments. We revise the manuscript by double checking for all notation usages and proving definition of them into table 1 as follows.

Reviewer 2: Concern #7:. Thereby, it is not easy to follow and understand the authentication scheme. Is it a cryptographic scheme?

Reviewer 2: Response #7: Thanks for the valuable comments. We revise the manuscript by adding briefly presentation used figure to easy understand the authentication scheme as follows.

----------------------------------------------------------------------------------------------------------------

Reviewer 3: Concern #1: Please describe the experimental results of this study in the abstract.

Reviewer 3: Response #1: Thanks for the valuable comments. We revise the manuscript by adding the result in terms of computation cost and communication costs in end of the abstract as follows.

“Finally, the computation cost of the proposed L-CPPA scheme in terms of message signing, single verification and batch verification is 694.161 ms, 60.118 ms, and 1348.218 ms, respectively. Meanwhile, the communication cost is 7757 bytes.”

Reviewer 3: Concern #2: The authors only compare with [48] (2019) and [49] (2020). The authors should compare with recent publications, e.g., [50] (2022) and other Lattice-based conditional privacy-preserving authentication schemes for VANET.

Reviewer 3: Response #2: Thanks for the valuable comments. This paper scopes and limitations of the research problem under only these schemes of [48] (2019) and [49] (2020) as mentioned as follows.

“The proposed L-CPPA scheme employs a fog server to generate and send a secret key for each registered vehicle via 5G-BS, which addresses the limitation arising in [52]. The TA in the proposed L-CPPA scheme saves its master private key to each fog server instead of the OBU of the vehicle, which addresses the limitation arising in [50, 51].”

Reviewer 3: Concern #3: The authors miss the Figure, "as shown in Figure ??", in Line 145 (Page 5).

Reviewer 3: Response #3: Thanks for the valuable comments. We revise the manuscript by adding figure to explain the proposed model as follows.

Reviewer 3: Concern #4: The authors should emphasize why their conditional privacy-preserving authentication scheme for fog computing with 5G-enabled VANET. Other schemes are also can be applied to fog computing with 5G-enabled VANET.

Reviewer 3: Response #4: Thanks for the valuable comments. We revise the manuscript by adding the research on how the proposed scheme outperforms other schemes in the related work section as follows.
