# Supplementary material for: L-CPPA: Lattice-based conditional privacy-preserving authentication scheme for fog computing with 5G-enabled vehicular system
Source: PLoS One. 2023 Oct 27;18(10):e0292690. doi: 10.1371/journal.pone.0292690 (PMC10610142; doi:10.1371/journal.pone.0292690)
Supplement: Attachment — Submitted filename: Response to Reviewers.docx [file pone.0292690.s002.docx]

Original Manuscript ID: PONE-D-23-11761R1

Original Article Title: “L-CPPA: Lattice-Based Conditional Privacy-Preserving Authentication Scheme for Fog Computing with 5G-Enabled Vehicular System”

To: PLOS ONE

Re: Response to reviewers

Dear Editor,

Thank you for allowing a resubmission of our manuscript, with an opportunity to address the reviewers’ comments.

Best regards,

Dr. Mahmood A. Al-Shareeeda.

Reviewer 2: Concern #1: The abbreviation for the proposed scheme is L-CPPA. However, LC-CPPA is appeared in page 8.

Reviewer 2: Response #1: Sorry for this mistake. Now the paper has revised this issue as follows.

(The following is an adversary model that the proposed L-CPPA technique should be able to counter.)

Reviewer 2: Concern #2: h3 is the third hash function, which should use subscript for 3 at pages 9 and 10.

Reviewer 2: Response #2: Thanks, we revised in page 9 as well page 10 by using subscript for 3.

Reviewer 2: Concern #3: PWD and TID are defined in Table 1. However, contents use PWD_i and TID_i instead of them.

Reviewer 2: Response #3: Thanks, we revise the table by adding same on the content of the paper.

Reviewer 2: Concern #4: T_1 is use for the timestamp. However, T_i is used for the definition of T_1 in page 11.

Reviewer 2: Response #4: we have define of ti in table to be I=1,2,3,n

Reviewer 2: Concern #5: Does the equation 6 require T_1 as a parameter for h_2 as the same as the equation 10?

Reviewer 2: Response #5: Sure and thanks, T_1 has been added now in the paper.

----------------------------------------------------------------------------------------------
