# Supplementary material for: L-CPPA: Lattice-based conditional privacy-preserving authentication scheme for fog computing with 5G-enabled vehicular system
Source: PLoS One. 2023 Oct 27;18(10):e0292690. doi: 10.1371/journal.pone.0292690 (PMC10610142; doi:10.1371/journal.pone.0292690)
Supplement: Attachment — Submitted filename: Response to Reviewers.docx [file pone.0292690.s003.docx]

**Original Manuscript ID:** PONE-D-23-11761R2

**Original Article Title: “** L-CPPA: Lattice-Based Conditional Privacy-Preserving Authentication Scheme for Fog Computing with 5G-Enabled Vehicular System”

**To:** PLOS ONE Editor

**Re:** Response to reviewers

Dear Editor,

Thank you for allowing a resubmission of our manuscript, with an opportunity to address the reviewers’ comments.

We are uploading (a) our point-by-point response to the comments (below) (response to reviewers), (b) an updated manuscript with yellow highlighting indicating changes (*Supplementary Material for Review*), and (c) a clean updated manuscript without highlights (*Main Manuscript).*

Best regards,

Al-Shareeda et al.

**Reviewer#1, Concern # 1:** Ensure that your manuscript meets PLOS ONE's style requirements, including those for file naming. The PLOS ONE style templates can be found at https://journals.plos.org/plosone/s/file?id=wjVg/PLOSOne_formatting_sample_main_body.pdf and https://journals.plos.org/plosone/s/file?id=ba62/PLOSOne_formatting_sample_title_authors_affiliations.pdf

**Author response:** Thanks for valuable comments.

**Author action:** We updated the manuscript by rewrite the paper by using LATEX format for POLS ONE journal.

**Reviewer#1, Concern # 2:** Add numbers to all sections and subsections (with respect to the journal template).

**Author response:** Thanks for valuable comments.

**Author action:** We updated the manuscript by adding numbers to all sections and subsections.

**Reviewer#1, Concern # 3:** Correct missed section’s references /numbers in the last paragraph of the introduction “Our remaining tasks are divided into several parts: Section proposes the 60 classification of the related works. Section provides the background of the vehicular 61 network. …” .

**Author response:** Thanks for valuable comments.

**Author action:** We updated the manuscript

**Reviewer#1, Concern # 4:** Check if all figures and tables are referenced and explained in the text.

**Author response:** Thanks for valuable comments.

**Author action:** We updated the manuscript by checking

**Reviewer#1, Concern # 5:** Check if all references respect the structure required by the journal and are alphabetically ordered.

**Author response:** Thanks for valuable comments.

**Author action:** We updated the manuscript by checking

**Reviewer#1, Concern # 6:** A more thorough proofreading of this manuscript is needed and the writing of the paper should be reviewed carefully. The followings are examples of typos to correct: At lines 13; 30 : " computing-based fog " do you mean : fog-based computing ? Line 70 : Several researchers [16–26] have been proposed PKI systems –-> Several researchers [16–26] have proposed PKI systems..Line 107 : sentence incomplete “Additionally, several researchers [41–47].” ; Al-Shareeda et al. [45] uses ECC ---> Al-Shareeda et al. [45] use ECC ; Line 139 : "Based on their knowledge," their it refers to whom? Do you mean "Based on our knowledge," Line 157 : “are the the 5G-Base” ---> “are the 5G-Base” Line 251: “the true identify” Do you mean “the true identity” Line 275: “should be not used” ---> “should not be used” Line 394: “If Eq. 12 oks” ---> “If equation 12 is verified” Line 399: “information i, Msgi ∈ [n].” do you mean “information Msgi, i ∈ [n].”? Line 431: “TIDi is the identify of the vehicle” ---> “TIDi is the identity of the vehicle” Line 443: “Once obtainin” ---> “Once obtained” “analyse/analyze (British / US English)”.

**Author response:** Thanks for valuable comments.

**Author action:** We updated the manuscript by revising all above concern on the manuscript

**Reviewer#1, Concern # 7:** Abbreviations should be explained during their first use: (line 78) TA is not previously explained.

**Author response:** Thanks for valuable comments.

**Author action:** We updated the manuscript by adding previously explained during their first use.

**Reviewer#1, Concern # 8:** The presentation of equation 11 should be reviewed and enhanced.

**Author response:** Thanks for valuable comments.

**Author action:** We updated the manuscript by enhancing the presentation of equation 11.
